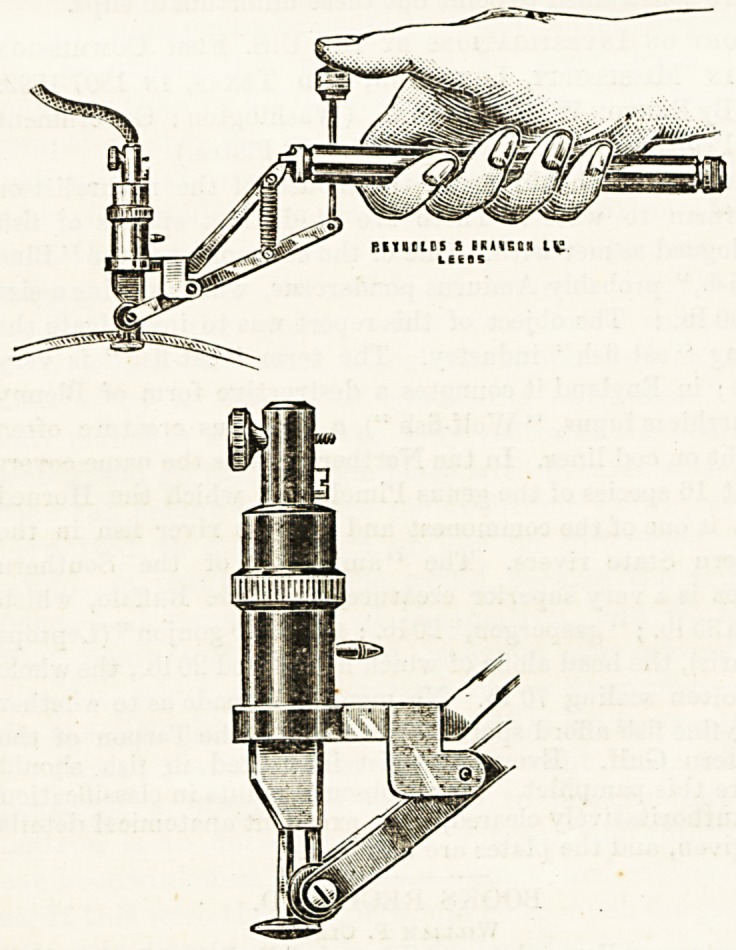# New Appliances and Things Medical

**Published:** 1899-12-30

**Authors:** 


### NEW APPLIANCES AND THINGS MEDICAL.

[We shall be glad to receive, at our Office, 28 & 29, Southampton Street, Strand, London, W.C., from the manufacturers, specimens of all new preparations and appliances which may be brought out from time to time.]

### NEW ELECTRIC OSTEOTOME.

(Reynolds and Branson, Limited, Briggate, Leeds.)

This new osteotome, which is worked by an electric motor, is the invention of Mr. Robinson, of Huddersfield. It is constructed somewhat on the lines of Cryer's instrument, but is the outcome of independent inventive genius. The osteotome, which is a modified drill, has a plain cutting edge of triangular bayonet shape. There is consequently no clogging with the *debris* of bone or soft tissues. The special advantage of the instrument is in the self-regulating action of the axle by means of a spring, which allows sufficient of the cutting-edge of the drill to act upon the bone when the button attached to the lever is pressed. When once the drill has made its groove in the bone and the wheels are in contact with the skull, the spring action renders the axle adaptable to the varying thicknesses of the bone, so that at all times a minimum of cutting-edge is exposed, and the button is kept closely applied to the inner surface of the skull, and thus the dura mater escapes injury. The spindle carrying the drill is hollow, to allow the flow of an antiseptic irrigating lotion for keeping the drill cool when in action.

### IMPROVED ELECTRIC GLOW LAMPS.

(Improved Electric Glow Lamp Co., 103, Queen Victoria Street, London, E.C.)

The conical silvered reflector lamp, which since its introduction has been most favourably received, may be regarded as the parent of the new half opaline lamp, a cheaper and more efficient variety, and the most recent development in reflecting lamps. In the manufacture of these lamps clear crystal bulbs are employed, but the upper half is coated with an opal glass mixture. This supplies a magnificent reflecting surface on the inner side, and at the same time permits of the passage of a certain proportion of well-diffused light through the opaline itself. The reflected light is soft and subdued, in contrast to the somewhat harsh glare of the metallic reflecting surface of the original prototype. The new opaline lamps certainly are a great improvement on those of earlier development, although, as far as general efficiency, economy, and durability are concerned, they appear to be no less satisfactory than the original "improved electric glow lamps" which, according to Dr. Hopkinson's report, are marked by superior efficiency as compared to other well-known lamps on the market. Thus the latest development of the Improved Electric Glow Lamp Company combines all the advantages of their earlier productions together with the pleasant and subdued effect of an opaline reflecting surface.

## Figures and Tables

**Figure f1:**